# Using an artificial neural network to map cancer common data elements to the biomedical research integrated domain group model in a semi-automated manner

**DOI:** 10.1186/s12911-019-0979-5

**Published:** 2019-12-23

**Authors:** Robinette Renner, Shengyu Li, Yulong Huang, Ada Chaeli van der Zijp-Tan, Shaobo Tan, Dongqi Li, Mohan Vamsi Kasukurthi, Ryan Benton, Glen M. Borchert, Jingshan Huang, Guoqian Jiang

**Affiliations:** 1 University of Minnesota, Minneapolis, MN 55455 USA; 2 School of Computing, University of South Alabama, Mobile, AL 36688 USA; 3 College of Allied Health Professions, University of South Alabama, Mobile, AL 36608 USA; 4 College of Medicine, University of South Alabama, Mobile, AL 36688 USA; 5 Qilu University of Technology (Shandong Academy of Science), Jinan, China; 6 Mayo Clinic, Rochester, MN 55905 USA

**Keywords:** Common data element, Artificial neural network, Schema mapping, Biomedical research integrated domain group (BRIDG) model

## Abstract

**Background:**

The medical community uses a variety of data standards for both clinical and research reporting needs. ISO 11179 Common Data Elements (CDEs) represent one such standard that provides robust data point definitions. Another standard is the Biomedical Research Integrated Domain Group (BRIDG) model, which is a domain analysis model that provides a contextual framework for biomedical and clinical research data. Mapping the CDEs to the BRIDG model is important; in particular, it can facilitate mapping the CDEs to other standards. Unfortunately, manual mapping, which is the current method for creating the CDE mappings, is error-prone and time-consuming; this creates a significant barrier for researchers who utilize CDEs.

**Methods:**

In this work, we developed a semi-automated algorithm to map CDEs to likely BRIDG classes. First, we extended and improved our previously developed artificial neural network (ANN) alignment algorithm. We then used a collection of 1284 CDEs with robust mappings to BRIDG classes as the gold standard to train and obtain the appropriate weights of six attributes in CDEs. Afterward, we calculated the similarity between a CDE and each BRIDG class. Finally, the algorithm produces a list of candidate BRIDG classes to which the CDE of interest may belong.

**Results:**

For CDEs semantically similar to those used in training, a match rate of over 90% was achieved. For those partially similar, a match rate of 80% was obtained and for those with drastically different semantics, a match rate of up to 70% was achieved.

**Discussion:**

Our semi-automated mapping process reduces the burden of domain experts. The weights are all significant in six attributes. Experimental results indicate that the availability of training data is more important than the semantic similarity of the testing data to the training data. We address the overfitting problem by selecting CDEs randomly and adjusting the ratio of training and verification samples.

**Conclusions:**

Experimental results on real-world use cases have proven the effectiveness and efficiency of our proposed methodology in mapping CDEs with BRIDG classes, both those CDEs seen before as well as new, unseen CDEs. In addition, it reduces the mapping burden and improves the mapping quality.

## Background

As Andrew Tanenbaum said: “The nice thing about standards is that there are so many to choose from” [1]. While Tanenbaum was talking about digital media standards, the statement applies to clinical data standards as well. Unfortunately, this truism has a corollary: the worst thing about data standards is that they require significant mapping efforts. More often than not, these are resource-intensive manual mappings.

In 2008 Rachel Richesson enumerated the problems with manual mapping, including the significant amount of time needed to develop and maintain the mappings; frequent lack of unambiguous, one-to-one mappings; and the context-specific nature of the mappings that limit their reuse [2]. Unfortunately, 10 years later these problems have yet to be fully resolved.

The experience of the Center for International Blood and Marrow Transplant Research (CIBMTR) regarding the implementation of electronic data capture and the adoption of data standards is an excellent case study of the problems with manual mapping. The CIBMTR is a research collaboration between the National Marrow Donor Program (NMDP)/Be The Match and the Medical College of Wisconsin. For more than 45 years, the CIBMTR has been collecting outcomes data and facilitating research in hematopoietic cell transplantation [3]. While the transplantation centers submit most of their data using a Web-based interface, the CIBMTR’s A Growable Network Information System (AGNIS) application [3] allows submissions directly from a transplantation center’s database to the CIBMTR. Common Data Elements (CDEs) from the National Cancer Institute’s (NCI) cancer Data Standards Registry and Repository (caDSR) form the foundation of data transmission via AGNIS. To either send or receive data using AGNIS, a transplantation center must map their internal data points to the CDEs [4].

While some transplantation centers and third-party vendors successfully use AGNIS, difficulties in manually creating and maintaining the mappings have limited its adoption [4]. To reduce the mapping burden, the CIBMTR developed a physical data model based on the Biomedical Research Integrated Domain Group (BRIDG) model [4] and mapped some of their CDEs to this model. In collaboration with multiple organizations such as the NCI and the Clinical Data Interchange Standards Consortium (CDISC) [5], the BRIDG model was developed to “produce a shared view of the dynamic and static semantics of a common domain-of-interest, specifically the domain of protocol-driven research and its associated regulatory artifacts” [6].

In addition to helping create a physical database model, the BRIDG model can facilitate mapping to other standards. For example, the BRIDG model has been harmonized and mapped to CDISC’s Clinical Data Acquisition Standards Harmonization (CDASH) and Study Data Tabulation Model (SDTM) [5]. As a result, the CIBMTR’s mappings to BRIDG can be used to facilitate mapping to CDISC. In 2016 mapping to CDISC became more critical when the Federal Drug Administration (FDA) mandated that most submissions to the FDA Center for Biologics Evaluation and Research (CBER) and Center for Drug Evaluation and Research (CDER) must comply with CDISC standards [7].

Whereas it can make mapping to other standards easier, mapping to BRIDG itself is difficult. Version 5.1 of the BRIDG model contains 320 classes [6]. While the model is subdivided into nine smaller subdomains [5], its size makes the mapping a significant challenge. The CIBMTR has mapped 1284 CDEs to the BRIDG model. This year-long mapping effort involved six subject matter experts, including a clinician and a BRIDG representative.

The CIBMTR has more than 2000 CDEs left to map. Based on the previous project, the mapping of the remaining CDEs will take approximately 2 years, which is unacceptable. One solution to reduce this mapping burden is to develop a semi-automated mapping tool that would recommend candidate matches from which a subject matter expert could select the best mapping.

Semi-automated mapping solutions are an area of active research, especially with ontology alignment. There are structural and conceptual similarities between CDEs and ontologies as both are associations of attributes/terms through relationships. This conceptual view shows the similarity of ontology alignment with the CDE to BRIDG mapping. There are two main methods for mapping ontologies: rule-based and learning-based. For the rule-based approach, a representative method developed by Noy and Musen [8] showcased a semi-automatic approach, PROMPT, based on the SMART algorithm of the same authors [9]. This approach first identifies label matching, then the user acknowledges or declines the merged entity pairs manually. Anchor-PROMPT [10] is an upgraded version of PROMPT that calculates the similarity based on the ontology structure.

For the learning-based approach, GLUE [11] uses machine learning techniques to do the ontology mapping. It uses two base learners, the Content Learner and Name Learner, as inputs to a meta-learner, which forms the final prediction; the meta-learner combines the weighted sum of the outputs of the base learners. The advantage is that it is a suited approach for textual instance descriptions. The disadvantage is that this approach is not applicable to relations or instances.

Other ontology matching algorithms exist. COMA [12] is a platform that combines the result of single matches. A statistical schema matching delivered by He and Chang [13] matches schemas by obtaining the generative model. Rubiolo et al. [14] present an approach based on an artificial neural network (ANN) model within a Knowledge Source Discovery agent. It helps the user to avoid unrelated search results and the possibility of making the wrong decision. Chortaras et al. [15] used a recursive neural network to learn the similarities between ontology concepts and then to combine two ontologies. This method has achieved some promising initial results. PRIOR+ [16] is an ontology mapping approach founded on propagation theory, information retrieval techniques, and artificial intelligence. It provides an estimate of f-measure for ontology constraint.

ANN methods are adopted in many of the algorithms mentioned above because ANNs have high accuracy, strong parallel processing ability, strong distributed storage and learning ability, strong robustness and fault tolerance to noise, full approximation of complex nonlinear relationships, and associative memory functions. Of particular importance, neural networks can extract features not available in many other machine learning methods.

Previously we developed an algorithm, Ontology Alignment by Artificial Neural Network (OAANN) [17, 18] to map two ontologies. It combines the benefits of rule-based and learning-based approaches to learn and adjust weights of concept name, concept properties, and concept relationships between a pair of concepts from two different ontologies. The algorithm applied gradient descent and the targeted design of each attribute similarity. We used this algorithm to successfully align concepts from two real-world biological ontologies with 0.9 Precision and 0.85 Recall, significantly reducing the time that domain experts spend on mapping ontologies.

This manuscript is an extension from our previously published work [19]. Compared with the original paper, most sections were significantly extended in this version. Major extensions (not including minor modifications) are summarized as follows. (1) Background: expanded introduction along with eight new references. (2) Materials and Methods: expanded description of the metamodels, especially the ISO 11179 CDE structure along with one new mapping example, four new figures, and some new references; added information about three new testing datasets along with one new table; and one new example for similarity determination. (3) Results and Discussion: extensive evaluation of our algorithm on real-world test cases; major modifications to the overall testing approach along with three new testing sets; one new flowchart describing the training process; one new sub-section about testing with preexisting mappings; one new chart summarizing the testing results; expanded description along with one new table in the Weights sub-section; and expanded description in the Overfitting sub-section.

Our most significant contribution in this work is to facilitate mapping two different meta-models: ISO 11179 CDEs and the BRIDG domain model. Importantly, as far as we know, no other work semi-automatically maps ISO 11179 CDEs to a domain model.

The rest of this paper is structured as follows. In the Materials section, we discuss key aspects of the ISO 11179 standard, the BRIDG model that form the foundation of the algorithm, and the datasets used for verification and testing. In the Methods section, we describe the details of our alignment algorithm. In the Results section, we report the testing of the algorithm and its application to a set of unaligned CDEs. In the Discussion section, we provide a detailed analysis of our experimental results. Finally, we conclude by discussing critical future work.

## Materials

### Metamodels and mappings

Our algorithm maps two different metamodels: ISO 11179-based CDEs and the BRIDG domain model. The ISO 11179 standard serves as the metamodel for the National Cancer Institute’s cancer Data Standards Registry and Repository (caDSR) [20]. This metamodel breaks a data point into reusable structures consisting of conceptual and representational components. The conceptual component refers to a Data Element Concept (DEC) [21]. The DEC consists of two parts: object class and property, describing concepts and concept characteristics, respectively.

**Fig. 1:**
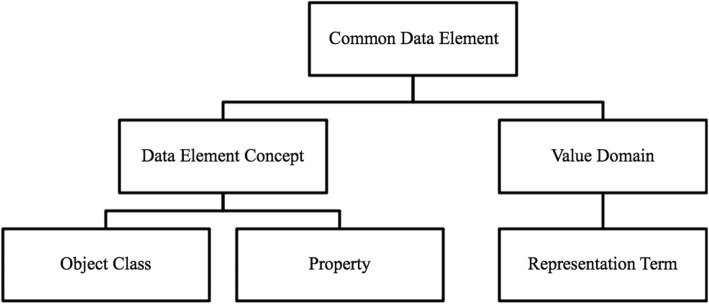
CDE structure. CDE mainly consists of two parts: Data Element Concept and Value Domain

**Fig. 2:**
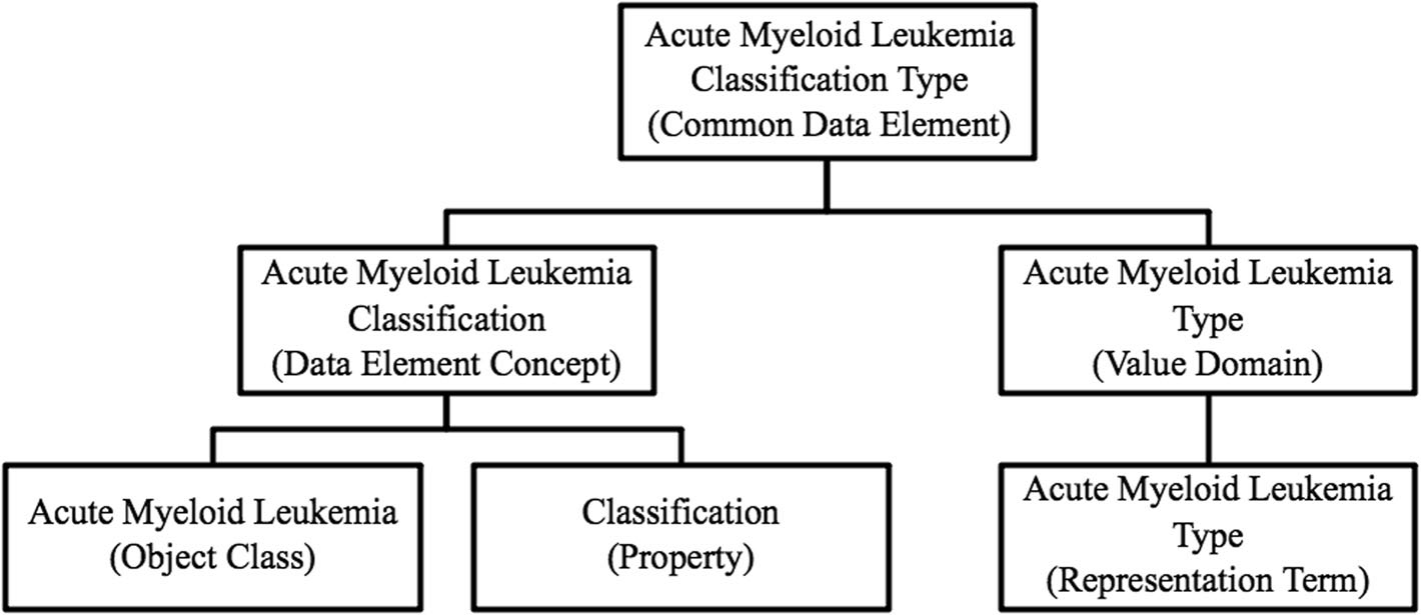
Example of CDE structure. The CDE structure for a data element capturing a patient’s specific type of Acute Myeloid Leukemia

The representational component of a CDE refers to a Value Domain (VD), which describes a set of allowed values in CDEs. The set of allowed values could be constrained to a specific set of permissible values or constrained by a list of requirements such as data type and maximum length [21]. Each VD has a representation term describing the information that VD is capturing. See Fig. 1 for an illustration of the structure. An example of a CDE is one used to capture a patient’s specific type of Acute Myeloid Leukemia. See Fig. 2 for the structure of the CDE Acute Myeloid Leukemia Classification Type [22].

The algorithm uses the six CDE attributes that capture the core semantics: CDE Long Name, Object Class, Property, Value Domain Long Name, Representation Term, and Question Text. Since the CDE Long Name should be created by concatenating the Object Class, Property, and Representation Term [23], it is one of the key attributes. For example, the CDE represented in Fig. 2 has an Object Class of “Acute Myeloid Leukemia”; a Property of “Classification”; and a Representation Term of “Acute Myeloid Leukemia Type.” Combined they produce a CDE Long Name of “Acute Myeloid Leukemia Classification Type.” The question text presents the semantics of the data element in everyday language instead of the formal syntax of the CDE. Therefore, it was included as an input variable. For example, the CDE in Fig. 2 has a Question Text of “What was the classification of the acute myelogenous leukemia?” The Value Domain Long Name was included to ensure the complete representation of the CDE’s semantics. CDE attributes such as allowed values, data type, and maximum length were not included in the analysis because they do not contribute to the semantic meaning of the CDE.

Within the caDSR, the constructs within a CDE and its attributes are associated with concepts from the NCI Thesaurus, a controlled vocabulary maintained by the Enterprise Vocabulary Service [20]. These concepts provide the concept name, definition, synonyms, and relationships to other concepts. Our algorithm currently leverages the concept name only.

**Fig. 3:**
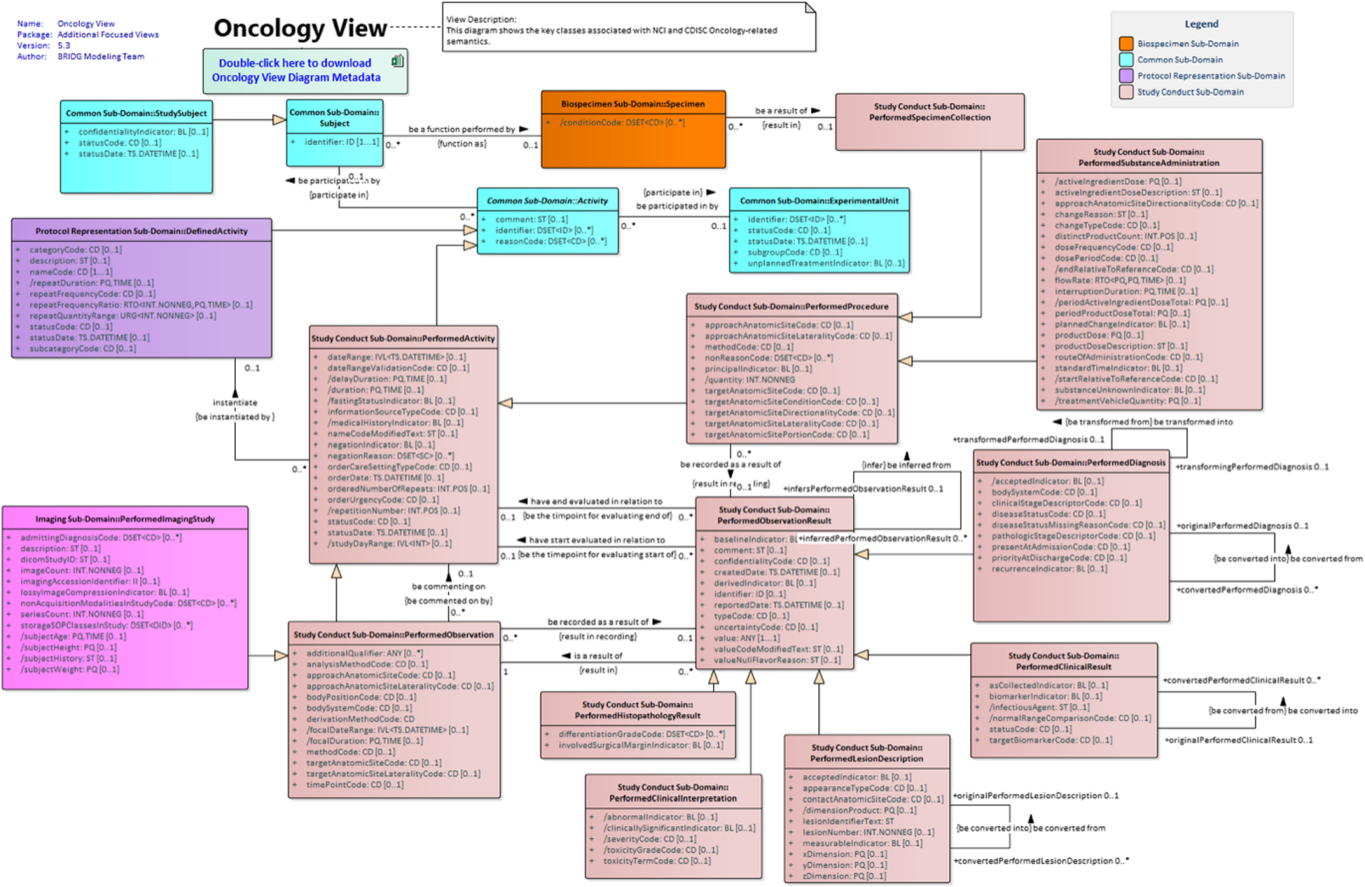
Oncology Subset of the BRIDG Model [6]. This is the Oncology view of the BRIDG model

In contrast to the ISO 11179, the BRIDG model represents data points using Unified Modeling Language (UML) classes, attributes, and relationships [24]. In UML, classes represent a classification of an object and attributes represent an object’s property [25]. In relation to the ISO 11179 metamodel, they correspond to the object class and property respectively [21]. To facilitate viewing of the BRIDG model, it has been subdivided into topic-specific views. Fig. 3 shows the Oncology view of the model.

The CDE for Acute Myeloid Leukemia Classification Type [22] is mapped to the BRIDG class “PerformedDiagnosis” and attribute “value.” 25 CDEs use a similar object class, property, and representation term structure to represent the specific disease classification. Of those 25, 17 have been manually mapped to the BRIDG model. All are mapped to the class “PerformedDiagnosis” and the attribute “value.” One can easily see how an algorithm can predict the mapping for the eight remaining CDEs. While this is a simple example, the algorithm leverages similar, though potentially subtle, patterns to map CDEs to BRIDG classes.

### Datasets

We used two different types of data sets: training sets and testing sets. The training set consisted of 1232 CDEs mapped to the BRIDG model. To create the base training set, we first examined the 1284 CDEs mapped to the BRIDG model by CIBMTR. These CDEs can be considered a “gold standard” because a robust team of experts, including a clinician and BRIDG representative, performed the mapping. Of the 1284 CDEs, 1232 are actively used and can be downloaded from the caDSR’s search site, the CDE Browser [26]; the remaining 52 CDEs were retired. Given that the 52 CDEs are retired (and not easily accessible to the broader community), it was decided to exclude them from the training set.

The 1232 active CDEs have been grouped into 57 BRIDG classes. We refined the training set to exclude those CDEs mapped to BRIDG classes associated with less than 10 CDEs. CDEs mapped to such BRIDG classes do not provide enough training examples and will interfere with the training results. The final training set consisted of 1134 CDEs mapped to 19 BRIDG classes. We divided the data into two groups: training and verification. We tested the effectiveness of the algorithm using different training to verification ratios: 90% training and 10% verification; 75% training and 25% verification.

For testing purposes, we compared the effectiveness of the algorithm against three different sets of CDEs that had been previously mapped to BRIDG. With these testing sets, the correct BRIDG class is known and can be compared to the prediction of the algorithm. Also, each testing set has different degrees of similarity to the training dataset. Since the bulk of the semantic meaning of a CDE is contained within the DEC [23] [21], we determined the degree of semantic similarity by calculating the percent of NCI Thesaurus concepts in the DECs of the testing set that also occurs in the DECs of the training set.

The first testing set consists of the previously mentioned 52 retired CDEs. Even though the retired CDEs are not actively used, their mappings to BRIDG are still valid. Furthermore, the same team mapped the retired CDEs as well as the training data. Hence, this represents the purest test case, as there are no subjective mapping differences that may lead to unexpected results. The second testing set consists of 220 CDEs that the CIBMTR mapped in 2017. These CDEs are associated with a new therapeutic domain and were mapped by a different individual and not the original mapping team. This represents a more difficult case, due to domain changes and change in the team. However, to this point, all the mappings were done in the same organization. The third set contains CDEs created by the NCI’s curation team to represent CDISC’s CDASH variables. Since the BRIDG release documentation contains mappings to the CDASH variables, mappings from these CDEs to the BRIDG model can be determined. Since the CDEs in this testing set were created and mapped by a different organization than the training set, this represents an excellent opportunity to see how the algorithm performs with a different organization’s content. As of December 2018, the NCI had created CDEs for more than 600 of the CDASH variables. However, not all of the CDEs had all of the attributes required by the algorithm nor were BRIDG mappings clear for all CDEs. We were able to use 186 CDEs for testing purposes.

The BRIDG model is periodically updated to reflect the changing clinical domain. The CDEs in the training set were mapped to version 3.0 of the BRIDG model, which was the current version in 2012. In 2017, version 5.0 of the model was released [6]. Since the new CDEs were mapped to BRIDG version 5.0, we tested only those CDEs mapped to BRIDG classes present in BRIDG 3.0. Table 1 summarizes the characteristics of each testing set.

**Table 1:** Similarity of testing data to training data

Testing Set	Number of CDEs	Semantic Similarity
Similar	52	86.54%
Moderately Different	220	68.64%
Different	186	4.52%

## Methods

### Purpose and overview of our method

Our method for mapping ISO 11179 CDEs to the BRIDG model is an expansion of an existing algorithm for aligning ontologies, Ontology Alignment by Artificial Neural Network (OAANN) [17, 18]. The ANN algorithm consists of training and verification phases. The goal of the training phase is to determine the best weights of the six attributes to classify the CDEs using the similarity of the CDE with each BRIDG class mapping. It outputs the top ten most probable BRIDG classes. Domain experts can use these recommendations to facilitate their mapping efforts. The verification phase verifies the accuracy of the mapping without changing the model.

### Data preparation

We determine the attribute similarity of two CDEs by first comparing the similarities between each of their six attributes. We determine the attribute similarity by first creating a matrix that calculates the similarity of each word in the attribute’s phrase. According to our previous research [17], we calculate string similarity using the following equation:

$$ {s}_{word}=1-\frac{d}{l} $$

**Fig. 4:**
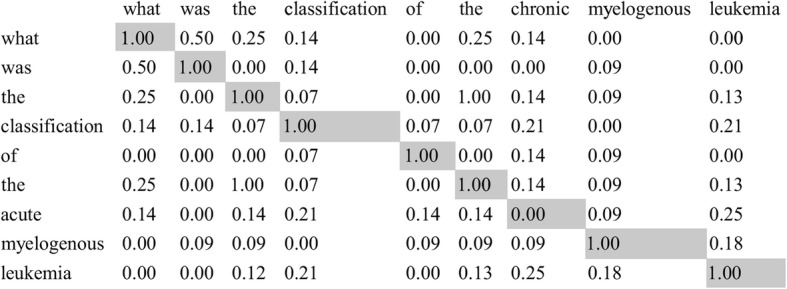
Example of word similarity matrix. This is the similarity matrix of the question text corresponding to the CDE Acute Myeloid Leukemia Classification Type and the CDE Chronic Myelogenous Leukemia Classification Type. Their corresponding question text is “What was the classification of the acute myelogenous leukemia?” and “What was the classification of the chronic myelogenous leukemia?” respectively. After calculating the similarity between every word and generating the word similarity matrix, we build the word similarity list by sorting and obtaining the maximum similarity from the matrix. The maximum similarity is represented by grey background. Note that after obtaining the maximum similarity, the similarities of this column and this row will be ignored, meaning that they will not participate in the sorting any more

The edit distance, a commonly used measure for measuring word difference, is denoted as *d*. The length of the longer string is *l*. For example, the edit distance *d* is two between word “what” and word “was”. The “h” in “what” is deleted in the first step. Then, “t” is substituted by “s” in “wat”. The above two steps successfully changed the word “what” into the word “was” by doing the minimum number of single-character edits, i.e. insertions, deletions or substitutions. The length of the longer word “what” is 4. Thus, the similarity between “what” and “was” is 0.5. We obtain the maximum similarity from the matrix and put the similarity into a list, *L*_*word* _ *similarity*_. We then delete the column and the row where the maximum similarity exists. We repeat this process until the matrix is empty and get the final list of *L*_*word* _ *similarity*_. A set of different word similarity threshold was chosen, i.e., from 0.6 to 0.9. These thresholds were applied both during the training and verification process. Once determined, the threshold does not change during the training and verification phases. We compare every similarity *l*_*n*_ from *L*_*word* _ *similarity*_ with the threshold we set. Two words match if *l*_*n*_ is greater or equals to the word similarity threshold. Finally, the attribute similarity *s*_*i*_(*i* = 1, 2…6) equals the number of words matched divided by the number of similarities in the word similarity list. Continuing the example from the Materials section, we compare the similarity of the CDE Long Names of the CDE Acute Myeloid Leukemia Classification Type [22] and the CDE Chronic Myelogenous Leukemia Classification Type [27]. Their question text is “What was the classification of the acute myelogenous leukemia?” and “What was the classification of the chronic myelogenous leukemia?” respectively. We build the word similarity matrix in Fig. 4. We sort the matrix and obtain the first maximum similarity from the first row and the first column and append this similarity into *L*_*word* _ *similarity*_. So *L*_*word* _ *similarity*_ = {1}. After repeating the process, *L*_*word* _ *similarity*_ = {1, 1, 1, 1, 1, 1, 1, 1, 0}. When we set the word similarity threshold to 0.9, the attribute similarity, *s*_6_ = 8/9.

After we obtain all attribute similarities, the overall similarity between two CDEs is calculated as the weighted sum of the attribute similarities, *s*_1_, *s*_2_, *s*_3_, *s*_4_, *s*_5_, and *s*_6_:

$$ s=\sum \limits_{i=1}^6\left({w}_i{s}_i\right) $$

where ${\sum}_{i=1}^6{w}_i=1$, *w*_*i*_ are initialized into 1/6 and were adjusted through the weight learning procedure.

We learn the weights for the six attributes during the group classification process. We design the learning problem as follows:

- Task, *T*: Recommend the most likely top ten BRIDG classes for a CDE.
- Performance measure, *P*: Accuracy measurements for the 1232 CDEs already grouped.
- Training experience, *E*: a set of classified CDEs by manual matching.
- Target function, *V*: a list of class recommendation.
- Target function representation: $V(b)={\sum}_{i=1}^6{w}_i{s}_i$.

### Network design

**Fig. 5:**
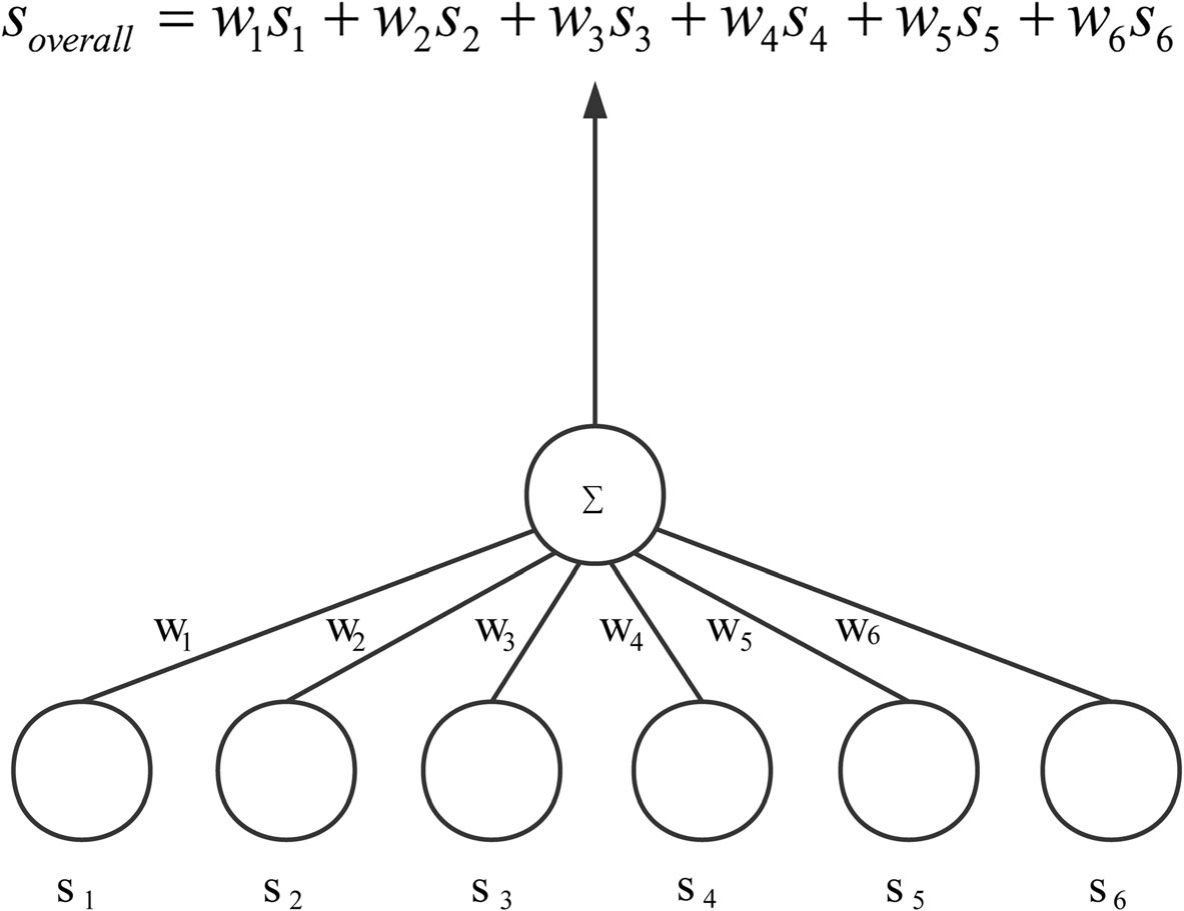
Neural network structure. The inputs are the similarities of six attributes. The output is the overall similarity between two CDEs

We modified our previous network design [17] to use a two-layer 6 × 1 network. Figure 5 illustrates the network’s vector inputs of *s*_1_, *s*_2_, *s*_3_, *s*_4_, *s*_5_, and *s*_6_. The neural network output is the overall similarity between two CDEs. Section B of the Method part gives the way to calculate the similarity value *s* between two CDEs.

### Hypothesis space and our searching strategy

The hypothesis space is a 6-dimensional space consisting of six vectors *w*_*i*_(*i* = 1, 2, ..., 6). We use gradient descent as our training rule. We minimize the training error of all training examples, so our task is to find such a vector. Training error *E* is calculated as

$$ E\left(\overrightarrow{w}\right)\equiv \frac{1}{2}{\sum}_{d\in D}{\left({t}_d-{o}_d\right)}^2 $$

in accordance with [28]. In $E\left(\overrightarrow{w}\right)$, the set of training examples is denoted as *D*, the target output for training example *d* as *t*_*d*_, the output of the network for *d* as *o*_*d*_. Two concepts are the base of training error, which are *s*_*avg*_ and *s*_*avg* _ *other* _ *cls*_.

- *s*_*avg*_: Let us say that *CDE*_m − *n*_ belongs to *BRIDG*_*m*_. From *BRIDG*_1_, we pick up the first element from *BRIDG*_1_ which is *CDE*_m − 1_ and calculate the overall similarity between the selected *CDE*_m − 1_ and other CDEs belong to this BRIDG class, separately. Then average these total similarities to get *s*_*avg*_.
- *s*_*avg* _ *other* _ *cls*_: We use the same CDE *CDE*_m − 1_, to calculate the similarity of this CDE with other CDEs from classes other than *BRIDG*_1_. Then average those similarities to get *s*_*avg* _ *other* _ *cls*_.

**Fig. 6:**
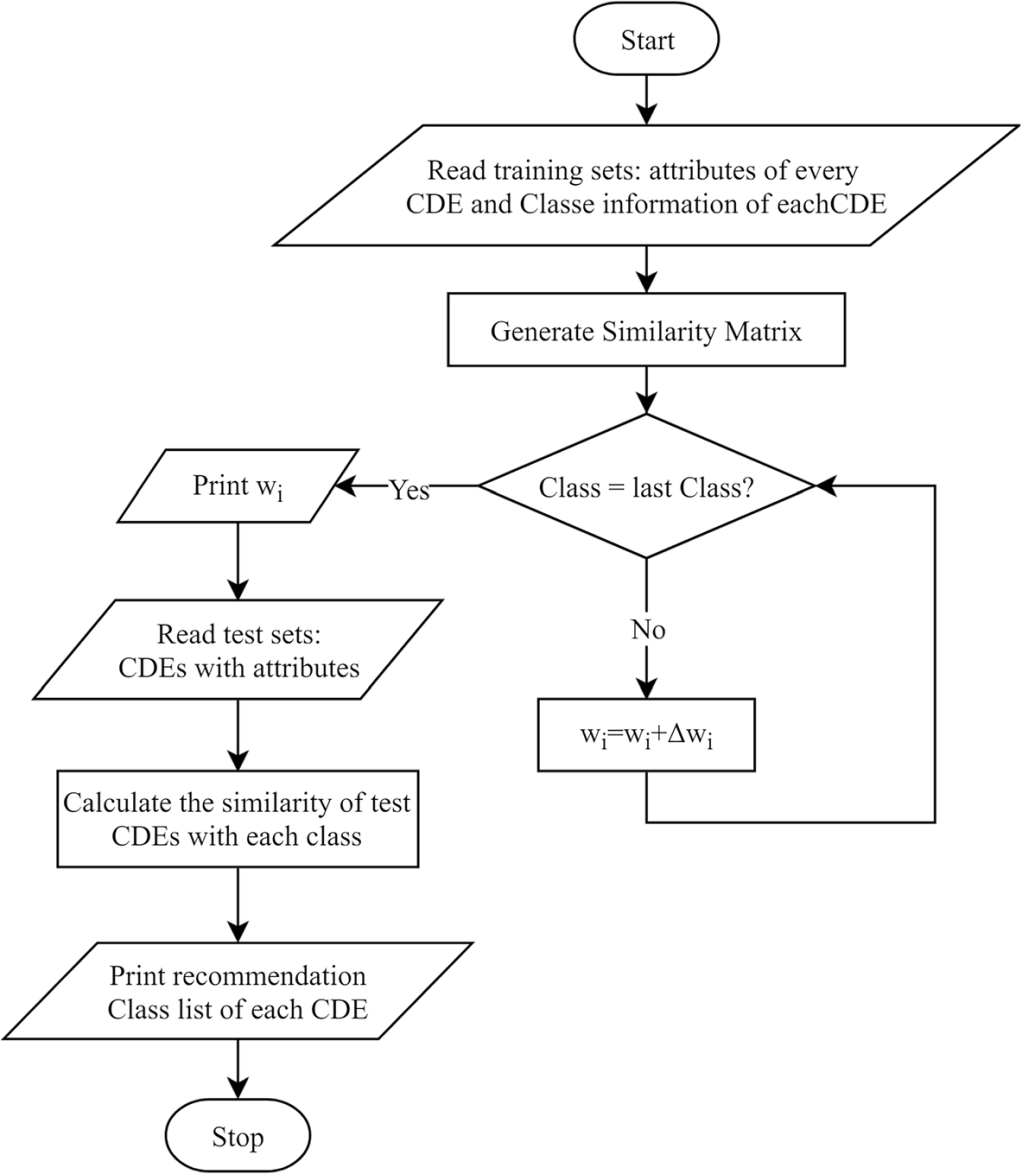
Training flow chart. The training flow demonstrates the process of training and recommendation

As we apply the gradient descent, we traverse one BRIDG class after another, pick up *CDE*_m − 1_ to calculate *s*_*avg*_ and *s*_*avg* _ *other* _ *cls*_, and finally make the adjustment of *w*_*i*_. The adjustment is represented by *Δw*_*i*_. The calculation of *Δw*_*i*_ is

$$ \varDelta {w}_i\equiv \eta {\sum}_{d\in D}\left({t}_d-{o}_d\right){s}_{id} $$

in accordance with [28]. *η* represents the learning rate of *Δw*_*i*_. The procedure is shown in Fig. 6. Notice that the BRIDG class is trained from less to more according to their CDE amount in each class. As the number of data increases, our accuracy rate generally increases.

### Error type

In order to determine if the weights need to be updated and how to update the weights, two different formulas are needed. For the former, we need to calculate the training error, which is given in the following formula:

$$ E\left(\overrightarrow{w}\right)\equiv \frac{1}{2}\sum \limits_{d\in D}{\left({s}_{\mathrm{max}}-{s}_{avg}\right)}^2 $$

We want our *s*_*avg*_ to be as large as possible to minimize $E\left(\overrightarrow{w}\right)$, so the larger value *s*_max_ should be the target output. As the goal is to minimize error, if the error is greater than zero and the number of updates are below a provided threshold, we update the weights using the following formula:

$$ \varDelta {w}_i\equiv \eta \sum \limits_{d\in D}\left({s}_{\mathrm{max}}-{s}_{avg}\right){s}_{id} $$

- *t*_*d*_ is represented by *s*_max_. *s*_max_ is the higher value between *s*_*avg*_ and *s*_*avg* _ *other* _ *cls*_.
- *o*_*d*_ is represented by *s*_*avg*_, which is the real output.

The weights are updated iteratively until verification accuracy stabilizes. As the iteration time (number of updates) increases, when the other parameters are using the same combination, the verification accuracy will increase and then gradually become stable. Experimentally, it was determined that when the iteration time is larger than 50, the error ($E\left(\overrightarrow{w}\right)$) will only fluctuate up and down by 1%. Since the results stabilized at this point, the maximum number of iterations was set to 50. Additionally, the learning rate *η* is fixed to 0.05. The value was chosen after performing some exploratory experimentation that indicated this value resulted in stable, repeatable results compared to higher values, and equivalent performance with respect to lower values.

Once the formulae were chosen, and the parameters fixed, we could then train the model to discover the six weights. Once the training is completed, we then proceed with the verification and testing results, which are discussed in Results.

## Results

### Overall testing approach

Our testing consisted of two phases. First, we verified the algorithm using a subset of the training data. Then, we tested the algorithm using three testing sets of CDEs for which we had pre-existing mappings. For each testing set, we determined the best match rate and the parameters needed to obtain the best match rate. We defined the match rate as the percent of total CDEs for which the existing BRIDG class mapped to the CDE was present in the list of top ten BRIDG classes returned by the algorithm. Table 2 shows the parameters used for verification and testing along with the optimal values as determined by our testing.

**Table 2:** Algorithm parameters

Parameter	Description	Values Tested	Optimal Values
Training ratio	Ratio of training to verification data	75% training and 25% verification 90% training and 10% verification	75% training and 25% verification
Training CDEs per BRIDG class	Determines the training list	4–10	8
Similarity threshold	Determines the threshold for considering two words to be similar	0.6–1.0	0.7 or 0.8

### Verification of training data

Table 3 shows the accuracy of the algorithm when it returns one to ten potential BRIDG classes for each CDE in the verification data set. When the training-verification ratio changes from 3:1 (75%/25%) to 9:1 (90%/10%), the training performance increases. When the algorithm returns more potential class matches, the accuracy of the algorithm increases and the performance differences between the 3:1 and 9:1 versions of the algorithm decreases. When the algorithm returns 10 potential class matches reaches ten, the accuracy of the algorithm reaches more than 90%. Also, we found that there was a significant increase in accuracy when the number of potential class matches returned increases from 1 to 2. This means the efficiency of the calculations and the cost performance of the results are relatively high. From the point of view from domain experts, returning 10 potential matches is reasonable because of the high accuracy.

**Table 3:** Accuracy with different training validation

Top n	Accuracy (training set: verification set = 3:1) (%)	Accuracy (training set: verification set = 9:1) (%)
1	33.99%	41.52%
2	51.96%	63.16%
3	64.71%	73.10%
4	71.90%	80.70%
5	76.14%	83.04%
6	82.03%	85.38%
7	85.29%	87.72%
8	86.93%	90.06%
9	89.22%	91.23%
10	92.16%	94.15%

### Testing with pre-existing mappings

Using the testing data sets described in the Materials section, we evaluated the algorithm to determine which combination of parameters produced the best match rate. Additionally, for each testing set, we ran the algorithm twice. One run utilized all CDEs contained in the testing set and the other run used only those CDEs that had been manually mapped to a BRIDG class for which there was sufficient training data. The goal of the last testing scenario was to determine the accuracy of the algorithm when we knew that it should produce an accurate match.

Overall, for the testing sets, we found that training-verification ratios of 3:1 (75%/25%) and 9:1 (90%/10%), performed equally well. However, the 3:1 version achieved this performance while requiring fewer training CDEs per BRIDG class (8 vs. 10). This makes the algorithm more flexible when testing novel CDEs. A similarity threshold of 0.7 or 0.8 achieved optimal match rates.

**Fig. 7:**
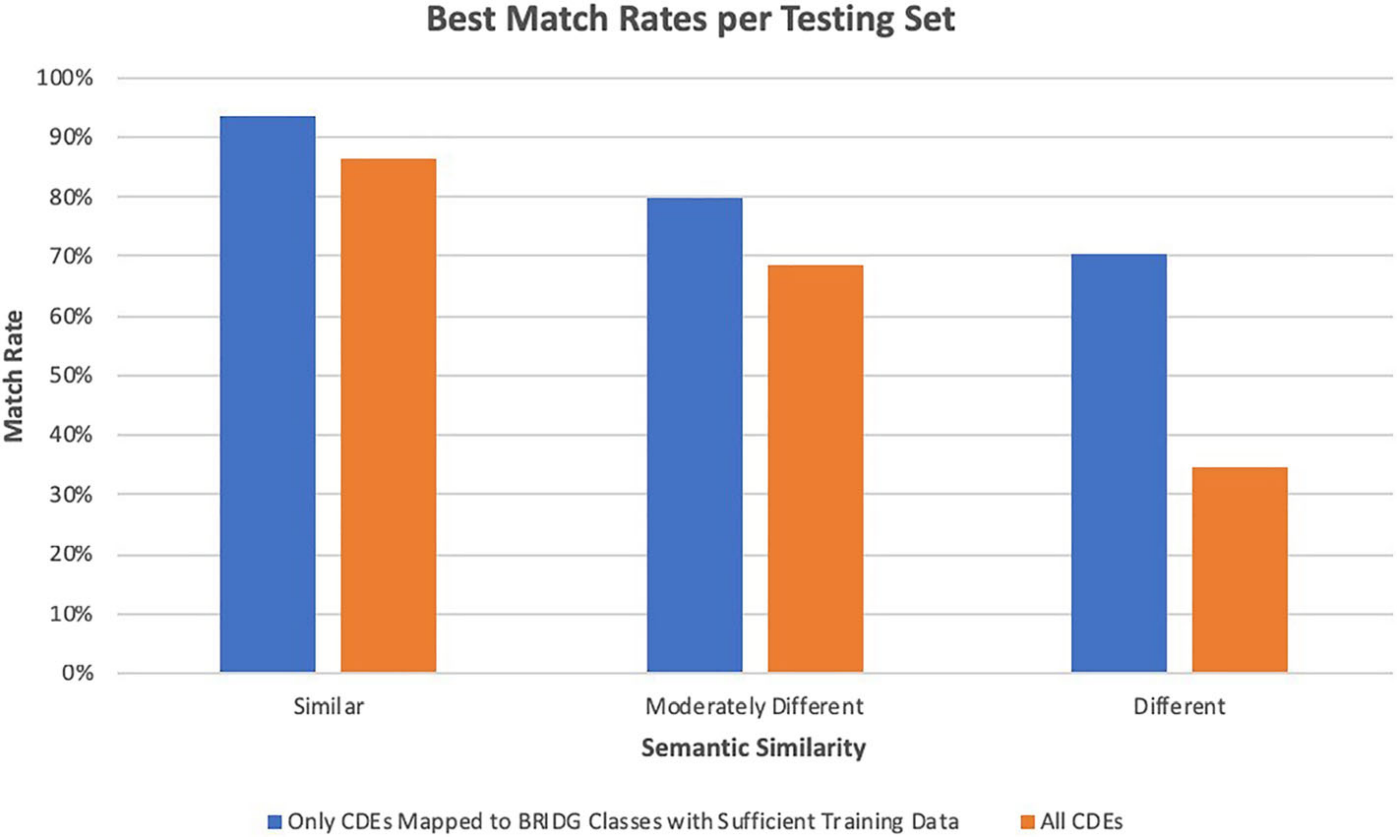
Best match rates per testing set. Bars refer to the best matching rate for testing sets with different degrees of semantic similarity compared to the training set: similar, moderately different, and different. The blue bars represent the situation in which the testing set contains only CDEs mapped to BRIDG classes with sufficient training data. The orange bars represent the situation that the testing set contains all CDEs

Depending upon the semantic similarity of the testing set and the availability of sufficient training examples, the algorithm produced match rates between 34 and 94%. The lowest match rates occurred when testing the algorithm using CDEs that were semantically different than the training set (4.52% similarity). Figure 7 summarizes the testing results.

## Discussion

### General result analysis

Fundamentally, the algorithm performs very well and has the potential to significantly reduce the mapping burden while improving the quality of the mappings. It should be noted that the use of our algorithm represents a semi-automated mapping process. While the algorithm can make suggestions, it will never replace the need for subject matter expert review and approval. While some testing scenarios resulted in lower match rates, the lower match rates are the result of two factors: semantic differences in the CDEs and lack of training data for the BRIDG class mappings. The testing results indicate that the greater the difference in the DEC concepts from the DEC concepts in the training set, the lower the overall match rate: 87% for the most similar dataset versus 34% for the most different when all CDEs in the testing set were tested. However, the match rate for all testing sets increased markedly when the algorithm was run against only those CDEs that mapped to a BRIDG class with sufficient training data: 94% for the most similar dataset versus 70% for the most different. This indicates that the availability of training data is more important than the semantic similarity of the testing set to the training set.

Increasing the size of the training set addresses both the semantic differences in the CDEs and the lack of BRIDG class instances in the training set. As the algorithm is used, and the appropriate mappings reviewed by a team of experts, the approved mappings can be added to the training set. This will incrementally improve the functioning of the algorithm. However, it is important to note that just adding data to the training set is not sufficient. One must add training data that increases the variability of the training set. When testing the Semantically Different testing set, we tried expanding the training data to include the Semantically Similar, and Moderately Different testing sets. This did not increase the match rate and, in one scenario, actually decreased the match rate. A closer examination of the expanded training set revealed that of the 38 BRIDG classes represented in the expanded training set, only five of them resulted in new training instances.

The algorithm can also assist with validating existing mappings. For example, the CDE Other Therapeutic Procedure Administered Indicator [29] was manually mapped to the BRIDG class “PerformedDiagnosis.” While the algorithm did match to this class, the ranking was ten. A closer review of the potential classes returned for this CDE showed that the second-ranked class “PerformedProcedure” was a better match. Indeed, of the six CDEs in the combined dataset that had a match with a rank of ten, half of the manual mappings were potentially incorrect, and the algorithm returned better potential matches.

### Weights

The weights are all significant in six attributes although they are slightly different from each other. This means that the attribute suggestion of the domain experts is accurate. We found that the weight for the Question Text is always slightly higher than the other attributes. Regardless of the parameters, from the training result, the average is 0.198, and the standard deviation is 0.010 for the initial 1232 CDEs. The average is 0.186, and the standard deviation is 0.001 for the 1504 combined CDEs. Since the Question Text is a complete sentence, it is more semantically useful and plays a greater role in classification. The second column of Table 4 contains an example of trained weights for the 1232 initial CDEs when Top n, iteration times, training-verification ratio, training elements in class, and similarity threshold is set to 10, 50, 9:1, 10, 0.7, separately. The verification of this combination is 159 out of 171, which is 92.98%. The third column of Table 4 contains an example of trained weights for the 1504 CDEs with the same parameters as the second column. From this combination, the verification is 187 out of 225, which is 83.11%.

**Table 4:** Example of attribute weights

Weight Name	Weight	
	Verification - 1232	Verification – 1504
*w*_1_ (CDE Long Name)	0.174620962	0.174501362
*w*_2_ (Object Class)	0.156449029	0.155271219
*w*_3_ (Property)	0.159148243	0.156401809
*w*_4_ (Value Domain Long Name)	0.159921116	0.164160267
*w*_5_ (Representation Term)	0.160018435	0.164215956
*w*_6_ (Question Text)	0.189842216	0.185449386

### Overfitting

One common problem encountered is overfitting, where the neural network picks weights tailored for the training instances versus the problem. We took two steps to address this. First, to ensure the team did not subconsciously introduce bias, CDEs were selected randomly instead of alphabetically.

Second, as previously noted, the ratio of training and verification samples had been modified. As seen in Table **3**, the changes in proportion had minimal impact for the larger values of n. For lower values, more training data led to improvements, indicating the learned weights are fitting to class concepts rather than instances. When the new CDEs are introduced, the 3:1 ratio became preferable as the new CDEs contain new classes. The improvement with the lower ratio indicates that the new classes vary from the initial set of classes; this indicates the 9:1 weights were learning the known class representations. A lower ratio allows more “flexible” weights, which handles new classes at the expense of some accuracy. This indicates that overfitting is not happening.

Finally, the results shown in Fig. **7** also provide evidence that overfitting problem is not a problem. The match rate for similar concepts is high, over 80% in both cases. Hence, the networks are learning concepts versus instances. The fact that the “moderately different” are again achieving well above random matching is indicative that the patterns the neural network are transferrable. Again, this is evidence that the networks are looking at patterns of behavior rather than particular concepts. The most interesting result is the “different” semantic concepts. When using CDEs that have sufficient training samples, the CDEs that have very different semantic meanings are achieving approximately a 70% match rate; this indicates the patterns discovered by the neural networks are highly transferable. It is only when all CDEs concepts are used in training data (even those with few examples) that we see a large drop. This is indicative that the neural network is (a) unable to extract what are the meaningful patterns for each type of CDE and (b) the low example CDEs are effectively introducing noise. While this leads to low match rates, it also demonstrates that the neural network is attempting to find meaningful, transferable patterns.

## Conclusions

The CDEs in the caDSR provide robust data point definitions that help ensure that clinical data adheres to the FAIR data principles: findable, accessible, interoperable, and reusable [30]. Mapping CDEs to the BRIDG model increases their value by providing a contextual framework and by facilitating their mapping to a variety of other data standards such as CDASH and SDTM.

Because manual mappings have many disadvantages including being extremely time-consuming and rather error-prone, there is an urgent need to map CDEs to BRIDG classes in a semi-automated manner. To handle this important challenge, we have developed an ANN-based machine learning algorithm that semi-automates the mappings between CDEs and BRIDG classes, followed by recommending a list of candidate classes to which the CDE of interest may belong. We evaluated our algorithm using a set of real-world use cases, and our experimental results showed that our algorithm has the potential to not only significantly reduce the mapping burden but also greatly improve the quality of the mappings.

In our future work, we plan to make several changes to the algorithm to further improve its effectiveness. Most significantly, we can leverage the wealth of information contained in the NCI Thesaurus ontology. Each CDE’s object class, property, and representation terms are created using concepts found within the NCI Thesaurus [20]. The NCI Thesaurus combines a reference terminology with an ontology to create a computable source of semantic information. In addition to providing a consistent naming convention and detailed definitions, the NCI Thesaurus also provides synonyms, semantic types, and relationships between concepts [31]. Expanding the algorithm to include this information will provide users with even more robust matching results.

We will also be adapting the algorithm to use the Data Element Concept (DEC) long name. We did not include the DEC long name in the algorithm because the caDSR tooling automatically constructs it by concatenating the Object Class and Property. Therefore, we assumed that Object Class and Property completely represent the semantics. However, 40% of the Data Element Concept Long Names in the training set were not an exact concatenation of the Object Class and Property. A future iteration of the algorithm should include the Data Element Concept Long Name.

There are some potential enhancements to the current training process that will be considered in the future. For instance, currently the training loss is considered as the sum of squared error, which may lead to an unnecessarily large gradient and possibly make the training process unstable under certain circumstances. To address this in future work, we may define the training loss as mean squared error. In addition, the problem may be defined as a multi-label classification problem, in which case binary cross-entropy may serve as a better loss measure. Moreover, using Stochastic Gradient Descent with momentum rather than the conventional gradient descent has been proposed for the future, as it can result in a smoother training process. Finally, we may also test the utility of Early Stopping, which stops the learning process if the error doesn’t decrease after a given number of epochs. This may make it more robust to inclusion of additional data, where 50 iterations may no longer be sufficient.

## Data Availability

All CDEs used for testing and training can be downloaded at: https://cdebrowser.nci.nih.gov/cdebrowserClient/cdeBrowser.html#/search. To find the CDEs, search for the following: Context: NCIP. Classification Scheme: Artificial Neural Network Algorithm for BRIDG Mapping. Classification Scheme Items: ● Training Set ● Testing Set - Semantically Similar ● Testing Set - Moderately Different ● Testing Set -Semantically Different Please note that search options must be adjusted to include CDEs that have been retired. The source code of the artificial neural network algorithm and the XML input files for all experiments can be downloaded at: https://github.com/Abclisy/ANN-in-CDE

## Abbreviations

AGNIS: A Growable Network Information System
ANN: Artificial neural network
BRIDG: Biomedical Research Integrated Domain Group
caDSR: Cancer Data Standards Registry and Repository
CBER: Center for Biologics Evaluation and Research
CDASH: Clinical Data Acquisition Standards Harmonization
CDE: Common Data Element
CDER: Center for Drug Evaluation and Research
CDISC: Clinical Data Interchange Standards Consortium
CIBMTR: Center for International Blood and Marrow Transplant Research
DEC: Data Element Concept
FDA: Federal Drug Administration
NCI: National Cancer Institute
NMDP: National Marrow Donor Program
OAANN: Ontology Alignment by Artificial Neural Network
SDTM: Study Data Tabulation Model
UML: Unified Modeling Language
VD: Value Domain
